# In Support of a Distinction between Voluntary and Stimulus-Driven Control: A Review of the Literature on Proportion Congruent Effects

**DOI:** 10.3389/fpsyg.2012.00367

**Published:** 2012-09-27

**Authors:** Julie M. Bugg, Matthew J. C. Crump

**Affiliations:** ^1^Department of Psychology, Washington University in St. LouisSt. Louis, MO, USA; ^2^Department of Psychology, Brooklyn College of CUNYBrooklyn, NY, USA

**Keywords:** cognitive control, proportion congruent, Stroop, flanker, voluntary control, stimulus-driven control

## Abstract

Cognitive control is by now a large umbrella term referring collectively to multiple processes that plan and coordinate actions to meet task goals. A common feature of paradigms that engage cognitive control is the task requirement to select relevant information despite a habitual tendency (or bias) to select goal-irrelevant information. At least since the 1970s, researchers have employed proportion congruent (PC) manipulations to experimentally establish selection biases and evaluate the mechanisms used to control attention. PC manipulations vary the frequency with which irrelevant information conflicts (i.e., is incongruent) with relevant information. The purpose of this review is to summarize the growing body of literature on PC effects across selective attention paradigms, beginning first with Stroop, and then describing parallel effects in flanker and task-switching paradigms. The review chronologically tracks the expansion of the PC manipulation from its initial implementation at the list-wide level, to more recent implementations at the item-specific and context-specific levels. An important theoretical aim is demonstrating that PC effects at different levels (e.g., list-wide vs. item or context-specific) support a distinction between voluntary forms of cognitive control, which operate based on anticipatory information, and relatively automatic or reflexive forms of cognitive control, which are rapidly triggered by the processing of particular stimuli or stimulus features. A further aim is to highlight those PC manipulations that allow researchers to dissociate stimulus-driven control from other stimulus-driven processes (e.g., S-R responding; episodic retrieval). We conclude by discussing the utility of PC manipulations for exploring the distinction between voluntary control and stimulus-driven control in other relevant paradigms.

## Introduction

Selective attention paradigms such as Stroop and flanker tasks contrast performance on incongruent (i.e., incompatible) trials where multiple responses are activated by a stimulus (e.g., naming the ink color of the word RED in blue ink; responding to the central arrow in <<<><<<) to congruent (i.e., compatible) trials where a single response is activated by a stimulus (e.g., naming the ink color of the word RED in red ink; responding to the central arrow in >>>>>>>). Interference effects emerge in such tasks with slowed (and sometimes more errant) responding on incongruent/incompatible trials relative to congruent/compatible trials. Although interference effects are routinely observed, their magnitude varies substantially as a function of theoretically important factors (e.g., working memory capacity, age, and clinical status). Of current interest is a factor termed proportion congruent (PC), referring to the proportion of trials that are congruent. PC dramatically modulates the size and even the direction (Logan and Zbrodoff, 1979) of the interference effect. Paradigms with mostly congruent trials (typically 67–80%) produce significantly larger interference effects than paradigms with mostly incongruent trials.

This review summarizes the growing literature on PC effects and examines the theoretically important question of what these effects signify about cognitive control. Part of the answer rests on careful consideration of the various ways that PC is manipulated. PC has been manipulated on three distinct levels: the list-wide level (e.g., separate blocks of trials are mostly congruent or incongruent); the item level (e.g., particular words are mostly congruent or incongruent); and the context level (e.g., items presented in one context are mostly congruent, but mostly incongruent in a different context). One goal of the review is to convince the reader that manipulations of PC at each level shed light on qualitatively different cognitive control processes. List-level control operates based on anticipatory information whereas item- and context-level control are rapidly triggered by the occurrence of particular stimuli or stimulus features. List-level PC manipulations index a more voluntary form of cognitive control, whereas item- and context-level PC manipulations index a reflexive or stimulus-driven form of cognitive control. In light of findings in the PC literature, a second over-arching goal of the review is to reconsider definitions of cognitive control, and we propose one that blends conventionally separate notions of controlled and automatic processes. Redefining cognitive control in this fashion suggests a need for new terminology to better describe the processes and representations affording control.

## The Many Faces of Cognitive Control

Cognitive control is by now a large umbrella term referring collectively to multiple processes that plan and coordinate behavior to meet task goals. According to convention, controlled processes are contrasted with automatic processes (Posner and Snyder, 1975; Shiffrin and Schneider, 1977). Controlled processes are voluntary, effortful, slow, and flexible. They prepare plans or task-sets that configure attention to selectively process task-relevant information during task performance. Automatic processes are involuntary, effortless, fast, and inflexible. They operate independently from controlled processes and may cause stimuli to capture attention or to retrieve associated responses. Strongly automatic processes are said to be cognitively impenetrable, or not under control. The controlled vs. automatic dichotomy has productively guided research in attention and performance over several decades. The PC literature has benefited from this distinction, but it has also produced new evidence challenging the dichotomy and conventional terminology by demonstrating that attentional control can occur in an automatic fashion. The oxymoronic term “automatic control” was coined by Jacoby et al. (2003) to describe these effects (p. 643). A contention of this review is that the controlled vs. automatic dichotomy should be abandoned and replaced by terminology that better characterizes the continuum between controlled and automatic processing (Bugg et al., 2008; Egner, 2008).

The terminology that we suggest here takes the general theory of attention and action (Norman and Shallice, 1986; Cooper and Shallice, 2000) as a starting point. Before elaborating on the major points we first describe some considerations about the concept of cognitive control that led us to adopt the terminology. The word control has different connotations for different researchers. For example, consider how cognitive vs. motor control differ.

Cognitive control refers to anticipatory, preparatory, endogenous, proactive, strategic, or voluntary processes that create, maintain, or adjust plans, task-sets, and attentional filters during performance. The spirit of this kind of control is top-down, supervisory, or executive in the sense that goals for performance are planned, monitored, and adjusted for success. In everyday life these control processes aid people in planning, thinking, and deciding on actions that will help them obtain their goals. For example, planning a driving route to run errands, focusing on a conversation with a friend in a crowded room, or surveying the field and choosing to pass to a teammate rather than an opponent in sports all rely on cognitive control processes.

Motor control refers to the processes and representations that coordinate actions. Current theories of motor control assume that motor schemas provide plans for action that are carried out by the motor system, and that online feedback from the environment and from internal simulations of the ongoing action can update and adjust movements to keep them in line with the action plan (Jordan and Rumelhart, 1992; Miall and Wolpert, 1996). Some aspects of motor control overlap with cognitive control. For example, like cognitive control, actions are planned, monitored, and adjusted by the motor system. As well, people have voluntary control of their actions. By contrast, other aspects of motor control, like the development of highly trained motor skills, overlap with the concept of automaticity. For example, the motor skills involved in driving a car are a common example of learned automatic routines. Many drivers have experienced arriving at an unintended destination like their place of work (when they originally planned a trip to the grocery store) as if they were driving on auto-pilot.

The differences between cognitive and motor control do not fit neatly into the controlled vs. automatic dichotomy, but instead speak to different levels of control. One important difference between levels is proximal vs. distal control. Proximal control refers to the representations, such as motor schemas, stimulus-response associations, and task-set representations that directly coordinate attention and action. Distal control refers to the control of proximal control; for example, by voluntary processes that select among motor schemas or task-sets, or as will be further developed in this review by exogenous cuing of proximal control representations.

Proximal control most closely resembles automatic processing. Automatic processes are commonly thought to be exogenous, involuntary, implicit, ballistic, reactive, cue/stimulus-driven, and cognitively impenetrable, or not under voluntary control; regardless, automatic processes are a fundamental component of control (cf. Hommel, 2007): they directly coordinate complex routine behaviors, and in this sense reflect proximal control of attention and action.

The interplay between proximal and distal control is insightfully framed by general theories of attention and action (Norman and Shallice, 1986; Cooper and Shallice, 2000). We outline the theory and consider its use for characterizing the multiple levels of control newly evidenced by the PC literature. The theory posits the supervisory and contention scheduling systems. The supervisory system is the distal controller, executive, or homunculus. This system “knows” current goals for action, and monitors the output of actions to ensure that goals are achieved. The system provides course correction and signals adjustments or new actions when the direction of performance has gone astray. The contention scheduling system is the workhorse and houses the proximal control representations or action schemas for familiar routines. Schemas refer to task and attentional sets, stimulus-response associations, and motor plans that provide the recipes for action needed to accomplish performance goals and sub-goals.

A fundamental assumption of the theory is that proximal representations are themselves controlled by either exogenous or endogenous means. Exogenous control refers to stimulus or cue-driven activation of associated proximal representations. For example, a coffee cup can trigger the motor movements needed to reach and grasp for the cup. Endogenous control refers to the supervisory system superseding ongoing activation of proximal representations that may lead performance astray. For example, a yellow traffic light could trigger a braking schema, but viewing an oncoming tailgater in the rear-view mirror could initiate supervisory intervention to inhibit the braking operation, and activate the schema for driving through an intersection to prevent an accident. Exogenous and endogenous forms of control are both distal in the sense that they act on the proximal representations that directly coordinate attention and action.

The theory highlights the terms proximal vs. distal control and exogenous vs. endogenous control and in doing so, preserves much of the spirit of the controlled vs. automatic distinction. Voluntary processes are capable of monitoring and adjusting attention and action, and stimuli are capable of triggering associated responses on a non-voluntary basis. In some sense the terminology simply re-casts “automatic” processes as proximal control, and “controlled” processes as those involved distally in the control of control. However, the theory also captures important nuances needed to explain emerging findings in the PC literature. For example, the conventional controlled vs. automatic dichotomy does not aptly describe situations where stimuli in the environment trigger adjustments to attentional filtering that occur in a rapid-online fashion and without awareness. Here proximal control is achieved through an attentional set that directly enacts attentional filtering operations; however, the activation of this attentional set is triggered exogenously by associated cues in the environment. Multiple lines of evidence for this kind of stimulus-driven control, which is subserved by stimulus-attention associations rather than stimulus-response associations, have emerged from the PC literature, and we advance the terms proximal vs. distal and exogenous vs. endogenous as tools for describing these effects in a common terminology.

Although these terms accommodate important themes in the controlled vs. automatic dichotomy, and do not “throw out the baby with the bathwater,” they also completely redefine automatic processes as being fundamental units of control (cf. Hommel, 2007). Automatic processes directly enact control over attention and action, and are distally controlled by endogenous and exogenous means. After reviewing the PC literature, we clarify these terms by distinguishing further between low (stimulus-response) and high (stimulus-attention) levels of proximal control, connecting the terminology to the range of PC phenomena, and discussing relations between levels of control more generally.

## Roadmap of the Review

A general aim of the PC literature has been to better understand the nature of the representations and processes controlling attention and action in selective attention tasks. Progress has been made in clarifying the nature of voluntary strategic processes that influence attentional selection, and stimulus-driven processes that control attentional selection and action. We review PC findings first in the Stroop literature, and then describe parallel developments in the flanker and task-switching literatures. We focus on list-wide proportion congruent (LWPC), item-specific proportion congruent (ISPC), and context-specific proportion congruent (CSPC) manipulations. Then we discuss processes and models that could explain the findings, and connect insights from the PC literature for understanding the many faces of cognitive control to the broader attention and performance literature.

## Stroop: LWPC, ISPC, and CSPC

The Stroop task involves naming the ink-color of a color word (Stroop, 1935). Identification times are faster for congruent trials (e.g., the word red in RED ink) than incongruent trials (e.g., the word red in Blue ink). The RT difference, termed the Stroop effect, reflects a failure of attention to filter out information from the distracting word. The size of the Stroop effect can measure the effectiveness of the attentional filter. A small Stroop effect indicates strong filtering of distracting information, whereas a large Stroop effect indicates weak filtering of distracting information. PC manipulations at the list-wide, item-, and context-specific levels modulate the size of Stroop effects and provide useful tools for measuring control-based attentional adjustments.

### List-wide proportion congruent manipulations

Many Stroop tasks present 50% congruent and 50% incongruent trials mixed at random. Consequently, participants are unable to accurately predict whether the next trial will be congruent or incongruent. LWPC manipulations vary the ratio of congruent and incongruent trials within a block. A mostly congruent block might be 75% congruent and 25% incongruent, and a mostly incongruent block the reverse. Stroop effects are larger for mostly congruent than mostly incongruent blocks, a finding termed the LWPC effect (e.g., Shor, 1975; Logan and Zbrodoff, 1979; Lowe and Mitterer, 1982; Logan et al., 1984; Cheesman and Merikle, 1986; Lindsay and Jacoby, 1994; West and Baylis, 1998; Kane and Engle, 2003).

Early accounts of LWPC effects assumed a role for strategic control. For example, using an ABOVE/BELOW spatial Stroop paradigm, Logan and Zbrodoff (1979) posited that participants strategically divide their attention between relevant and irrelevant dimensions, weighting the irrelevant dimension more heavily than the relevant in mostly congruent than mostly incongruent blocks (see also Logan, 1980; Logan et al., 1984, for evidence with color-word Stroop; Lowe and Mitterer, 1982). This is because the irrelevant dimension tends to validly cue the “value” of the relevant dimension (i.e., the response) in a mostly congruent list. In a similar vein, the dual-mechanisms of control account posits that participants develop expectancies about upcoming trials and modulate control proactively (e.g., Braver et al., 2007). When participants expect a congruent trial, as in a mostly congruent list, they may voluntarily pay more attention to the word, which usually corresponds to the correct response. Such a strategy would speed processing of congruent items, create strong interference for incongruent items, and increase the size of the Stroop effect. When incongruent trials are expected, participants may double-down on their attempt to filter out word information (i.e., avoid word reading). This strategy would slow down identification for congruent items (as the word would have less of a facilitating effect), speed up identification for incongruent items (less interference because of better attentional filtering), and decrease the size of the Stroop effect. Such predictions have been confirmed in some studies (e.g., Logan and Zbrodoff, 1979; Kane and Engle, 2003, Experiment 4; West and Baylis, 1998). Moreover, Lindsay and Jacoby (1994) have provided evidence from a process-dissociation procedure showing that the color-naming process (representing attention to the relevant dimension) does not vary as a function of LWPC. Rather, manipulating PC in this fashion produces a selective effect on the word reading process (representing attention to the irrelevant dimension). These data also point to a strategy that filters words differentially for mostly incongruent and mostly congruent lists.

Although strategic explanations of the LWPC effect are both parsimonious and intuitive, there has been much recent debate over the kinds of processes that may account for PC modulations to Stroop, including the LWPC effect. Not all accounts suggest use of a mechanism that relies on information about the list (i.e., the likelihood that the irrelevant dimension will be valid; the likelihood that trials will be incongruent) to strategically alter attention in advance of stimulus presentation. A competing account attributes LWPC effects to item-specific mechanisms (Bugg et al., 2008; Blais and Bunge, 2010) that operate only after a stimulus has been presented, and rely on information about particular stimuli. Before fully considering this account, we describe such item-specific mechanisms.

### Item-specific proportion congruent manipulations

A formative innovation was to manipulate PC at the level of individual items, rather than at the list-wide level (Jacoby et al., 2003). An ISPC manipulation assigns different PC levels to different sets of items. In the seminal study, Jacoby et al. (2003) assigned particular words to be mostly congruent or mostly incongruent. For example, the words RED and WHITE could be 80% congruent and 20% incongruent, whereas the words BLACK and GREEN could be 20% congruent and 80% incongruent. The mostly congruent and mostly incongruent items were randomly intermixed, resulting in a LWPC of 50/50 congruent and incongruent trials. Thus, participants were unable to predict whether an upcoming trial would be congruent or incongruent. That is, there was no basis for participants to form a list-wide strategy to increase word reading or filter out words. Still, a PC effect was observed indicating significantly less interference for mostly incongruent than mostly congruent items. Jacoby et al. termed this the ISPC effect, and firmly established that not all PC effects depend on having advance information about PC such as list-level information. In the ISPC paradigm, a participant could not know whether the word on a given trial was from the mostly congruent or mostly incongruent set until it was presented. As such, implementing a list-wide strategy to increase or prevent word reading would have been non-optimal (indeed, the fact that similar Stroop effects were *not* obtained for both item-types shows that such a strategy was not used).

Jacoby et al. (2003) suggested that ISPC effects may reflect rapid, online, stimulus-driven control over attentional filtering – a kind of oxymoronic “automatic control” (p. 643). On this view, individual items become associated with the attentional filters that are frequently employed for their respective item-types during the experimental session. For example, mostly congruent items become associated with an attentional filter that weakly filters word information, and mostly incongruent items become associated with an attentional filter that strongly filters word information (cf. Trainham et al., 1997; Jacoby et al., 1999). When an item appears as a stimulus on-screen it reflexively triggers the retrieval of its associated attentional filter, and this filter rapidly adjusts current attention settings to provide online control over processing of the Stroop item. Using the sample stimuli above, the idea is that when the word BLACK is presented, processing of the word is quickly attenuated. By contrast, when WHITE is presented, it triggers fuller processing of the word. In other words, the influence of the word is controlled at the item level, with the item itself acting as the environmental cue to enact a particular attentional set. Consistent with this view, process-dissociation estimates indicated that, like the LWPC manipulation, the ISPC manipulation was associated with a change in the contribution of the word process, and no change in the contribution of the color process across mostly incongruent and mostly congruent items (Jacoby et al., 2003).

An alternative view of the ISPC effect centers on an item-specific associative learning mechanism that capitalizes on the frequency with which particular words and colors are paired in ISPC designs (Jacoby et al., 2003). ISPC manipulations introduce item-frequency as a confound and ISPC effects could reflect that participants learn to respond faster to high than low frequency word-color pairs (Logan, 1988). Mostly congruent item-types repeat specific congruent items frequently and specific incongruent items infrequently (sometimes never repeated in a single block). By contrast, mostly incongruent item-types may repeat specific incongruent items frequently and specific congruent items infrequently. As such, the ISPC effect may reflect speeded responding for high-frequency items. In a similar vein, Schmidt and Besner (2008) suggested that because PC is confounded with contingency, a stimulus-response contingency-learning process may account for the ISPC effect. By their contingency account, the reason participants are faster in responding to congruent trials for mostly congruent than mostly incongruent items, and in responding to incongruent trials for mostly incongruent than mostly congruent items, is not due to item-specific control. Rather, they purport that participants learn the correlations between particular words and colors (cf. Musen and Squire, 1993; Dishon-Berkovits and Algom, 2000; Melara and Algom, 2003) and use the word to predict high contingency responses (colors). Again using the sample stimuli above, the idea is that participants learn to say “green” whenever BLACK is presented and “white” whenever WHITE is presented. The contingency account contends that the ISPC effect is entirely due to these contingency-learning processes and attentional modulation based on PC (i.e., item-specific control) plays no role in the effect.

#### Disentangling item-specific control and contingency learning

Item-specific control and contingency-learning accounts of the ISPC effect both assume a stimulus-driven control process, however they differ on the nature of the proximal representations enacting control. The contingency-learning account assumes that stimulus-response associations are the representation controlling action. The item-specific control account further assumes stimulus-attention associations: stimuli are associated with self-tailored attentional sets triggering rapid-online filtering of irrelevant information. There has been much debate in the literature over the contribution of item-specific control and item-specific contingency learning to ISPC effects. This question has been addressed in three ways: (a) by providing direct empirical evidence for the contingency account, and by crafting (b) item-specific designs, and (c) higher-order context-specific designs that rule out, or control for the influence of learning stimulus-response contingencies.

The primary evidence in favor of the contingency-learning account stems from Schmidt and Besner (2008, but see also Schmidt et al., 2007; Hutchison, 2011; Atalay and Misirlisoy, 2012) who re-analyzed Jacoby et al. (2003) to de-confound PC and contingency. Instead of conducting the standard analysis that compares Stroop interference for mostly congruent items (i.e., incongruent-congruent) to mostly incongruent items (i.e., incongruent-congruent), they used a contingency analysis to contrast interference for items that were equated in contingency (e.g., high contingency trials: mostly incongruent-incongruent – mostly congruent–congruent; low-contingency trials: mostly congruent incongruent – mostly incongruent-congruent). They predicted and confirmed that the contingency analysis would yield main effects of trial type and contingency but no interaction. According to Schmidt and Besner, the absence of the interaction was a key piece of evidence countering the item-specific control account, because accounts emphasizing modulation of word reading would predict “incongruent trials should be more affected by attention, given that the majority of the Stroop effect is interference with little or no facilitation from congruent trials” (p. 516).

Although Schmidt and Besner (2008) provided strong evidence in favor of the contingency account, Bugg et al. (2011a) questioned the ubiquity of the account and whether ISPC effects are always dominated by contingency learning. Their design de-confounded PC and contingency and permitted examination of the ISPC effect using the standard analysis approach. The key design feature was designating the relevant (to-be-named) dimension as the signal of ISPC rather than the irrelevant dimension, which was used in prior studies (e.g., Jacoby et al., 2003; Schmidt and Besner, 2008). When the irrelevant word dimension predicts ISPC, words signal *both* information that could be used to modulate word reading, and the most frequently paired response. When the relevant color dimension signals ISPC, contingency is equated across all four cells (combining PC and trial type) because the relevant dimension is 100% predictive of the correct response in each cell. Per a contingency account, an ISPC effect should not be obtained in this design because only PC (and not contingency) differentiates mostly congruent and mostly incongruent items. According to the item-specific control account (Bugg et al., 2011a), an ISPC effect should be obtained because participants use information signaling PC to modulate reliance on the word dimension.

In the critical experiment providing support for the item-specific control account, Bugg et al. (2011a, Experiment 2) found a significant ISPC effect using the above design in a picture-word Stroop task (“Name animal in picture, ignore word”). Moreover, the ISPC manipulation had a selective influence on incongruent trial performance with RTs speeded for the mostly incongruent than mostly congruent items, a finding consistent with Schmidt and Besner’s (2008) prediction that a control mechanism would have a stronger influence on incongruent trials. In addition, Bugg et al. examined whether participants would transfer the control settings associated with mostly incongruent and mostly congruent items to a new set of stimuli. Importantly, these stimuli were new exemplars from the four animal categories that comprised the relevant dimension for training trials in the first two blocks of the task. For example, pictures of birds and cats were mostly congruent during training and pictures of dogs and fish were mostly incongruent during training. During the third block new pictures of birds, cats, dogs, and fish were presented as transfer trials and importantly these transfer trials were 50% congruent. Thus, if an ISPC effect was obtained for the transfer trials, it would suggest that participants had applied the control settings they associated with the training trials to these new transfer items. Indeed, transfer was shown. These findings are theoretically important because they challenge the contingency account, and other frequency-based accounts (e.g., Logan, (1988) that predict a RT advantage not only for mostly incongruent-incongruent trials (as was found) but also for mostly congruent-congruent trials, which was not observed.

So, where does that leave us? There is clearly evidence supporting both the contingency account and the item-specific control account. Such patterns mirror the original conclusion of Jacoby et al. (2003) who suggested a role for both processes. While such a conclusion is reasonably satisfying, it is important to understand the conditions under which one vs. the other dominates. For example, it would be prudent for researchers interested in stimulus-driven *control* to employ the design used by Bugg et al. (2011a, Experiment 2) rather than Jacoby et al. (2003). Bugg et al. proposed the basis of the ISPC signal as a design principle to differentiate ISPC designs producing effects reflecting cognitive control vs. contingency learning. When the relevant dimension signals ISPC, ISPC effects are control-based (see Bugg et al., Experiments 1 and 2 for support), but when the irrelevant dimension signals ISPC, effects are contingency-based. In support of the latter, it was found that when the exact same design was used as in Experiment 2, but words were designated mostly congruent or mostly incongruent, an ISPC effect was obtained but all of the action was in the congruent trials, consistent with predictions of a contingency account (Bugg et al., Experiment 3).

Color-word Stroop purists might contend that evidence for item-specific control in picture-word Stroop does not imply item-specific control in color-word Stroop. Picture-word and color-word Stroop effects may tap different processes (but see van Maanen et al., 2009). For example, Dell’Acqua et al. (2007) examined the locus of the interference effect in both Stroop tasks using a psychological refractory period paradigm, and found that picture-word Stroop interference arises earlier than color-word Stroop interference. In picture-word Stroop, the locus is the perceptual encoding stage whereas in color-word Stroop the locus is the response selection stage. Given that interference may serve as a trigger for item-specific control (e.g., Blais et al., 2007; Braver et al., 2007), it is possible interference arises too late in color-word Stroop paradigms for item-specific control to effectively modulate the influence of the distracting word. Countering this concern, Bugg and Hutchison (2012) replicated the critical patterns supporting the role of item-specific control in the ISPC effect using a color-word Stroop paradigm. That is, they showed that when the relevant dimension (here, color) signaled ISPC, effectively eliminating the confound between PC and contingency, an ISPC effect was still obtained contrary to the contingency account. In addition, like the patterns observed in picture-word Stroop (Bugg et al., 2011a, Experiment 2), the ISPC effect selectively influenced performance on the incongruent trials and transfer of item-specific control settings was observed for novel 50% congruent trials that consisted of “old” mostly congruent and mostly incongruent colors paired with new words. These findings provided further support for the idea that the locus of the ISPC signal (relevant vs. irrelevant dimension) is an important factor moderating use of item-specific control vs. item-specific contingency learning. However, in another experiment, Bugg and Hutchison showed that this view may be overly simplified; signaling ISPC via words can also produce control-dominated effects.

The goal of that experiment was to return to the original design of Jacoby et al. (2003) where words signal ISPC and the confound between PC and contingency is present to determine whether there are limitations on use of contingency learning in such designs. Bugg and Hutchison (2012) hypothesized that evidence favoring the contingency account would be limited to a two-item set design. In a two-item set design, the design that was used by Jacoby et al. (2003, Experiments 2a, 2b, and 3) and Schmidt and Besner (2008) in formulating the contingency account, a single high contingency response exists for both the mostly congruent and mostly incongruent word sets. In the mostly congruent set, it is the congruent response and in the mostly incongruent set, it is the incongruent response associated with the opposite color in that set. Contrast this with a four-item set where a single high contingency response option exists for the mostly congruent set but does not exist for the mostly incongruent set. There is no high contingency incongruent response. Rather, there are three equally probable responses on incongruent trials. This means that participants cannot predict with high accuracy the response that is mostly likely on any incongruent trial during a task that employs a four-item set. Given these differences, Bugg and Hutchison predicted that although the word signals ISPC in both a two- and four-item set, contingency-learning mechanisms would dominate only in the two-item set.

Two approaches were used to determine the underlying mechanism(s) responsible for the ISPC effect in the two- and four-item sets. The first was to examine the ISPC pattern (Bugg and Hutchison, 2012). For the two-item set, a symmetrical pattern was obtained reflecting speeding of RT on congruent trials from the mostly congruent set and on incongruent trials from the mostly incongruent set, the two trial types for which a high contingency response existed. By contrast, a stronger effect of the ISPC manipulation was found for incongruent trials than congruent trials in the four-item set. In particular, the RT speeding on incongruent trials in the mostly incongruent as compared to the mostly congruent set was larger than the speeding on congruent trials in the mostly congruent as compared to the mostly incongruent set, a pattern that is similar to the control-based ISPC pattern obtained in prior studies (Bugg and Hutchison, 2012, Experiments 1 and 2; Bugg et al., 2011a, Experiments 1 and 2).

The second approach was to examine transfer performance in the two- and four-item sets (Bugg and Hutchison, 2012). Transfer was assessed by presenting “old” mostly congruent and mostly incongruent words paired with new colors in a final block of trials, and these transfer items were 50% congruent. Per a contingency account, transfer should not be obtained because participants have no prior experience predicting/naming the new transfer colors. Per an item-specific control account, transfer should be obtained if participants have learned to use the word to modulate attentional settings because the old mostly congruent and mostly incongruent words still appear on transfer trials. For the two-item set, no evidence of transfer was obtained. That is, the magnitude of interference was similar for the mostly congruent and mostly incongruent words presented in new colors. By contrast, an ISPC effect was observed for the transfer items in the four-item set. Here, less interference was observed when responding to new colors that were paired with words from the mostly incongruent set than with words from the mostly congruent set. The selective effect of transfer in the four-item set, in conjunction with the ISPC pattern itself, is consistent with the view that item-specific control dominated in the four-item set. Participants utilized the word as a signal of control, quickly attenuating its influence when the word was mostly incongruent, and more fully processing the word when the word was mostly congruent. These findings suggest an update to the item-specific control account in showing that contingency-learning mechanisms do not always dominate when words are the signal of ISPC. Rather, contingency learning appears to dominate under select conditions, such as when a two-item set is used and high contingency responses can be learned for both congruent and incongruent trials.

#### List-wide proportion congruent manipulations: revisited

Previously, we mentioned that some folks have posited accounts of the LWPC manipulation that are not based on a strategic control process that prepares attention in advance of stimuli, but instead reflect the operation of stimulus-driven mechanisms such as item-specific control and item-specific contingency learning (e.g., Bugg et al., 2008; Schmidt and Besner, 2008; Blais and Bunge, 2010). The possibility that LWPC manipulations trigger use of item-specific mechanisms is bolstered by the fact that LWPC is perfectly confounded with ISPC in the standard design used in LWPC studies. Mostly congruent lists are composed from stimuli that are mostly congruent at the item level. For example, if four stimuli are used, each one is presented 75% of the time in a congruent color making for an ISPC of 75% congruent. In a mostly incongruent list each of the four stimuli are presented 25% of the time in a congruent color, such that the stimuli have an ISPC level of 25% congruent. Thus, participants could be modulating word reading on an item-by-item basis rather than employing a global and sustained word reading (or word avoiding) strategy. Similarly, participants could rely on item-specific contingency learning, predicting the responses that are mostly likely for particular words upon their presentation.

An initial hint in the literature that the latter type of mechanism may be contributing to the LWPC effect was evident in one of the earliest studies on the effect. Logan et al. (1984) found that the LWPC effect was robust when two word-color contingencies were present in the lists (Experiments 1 and 2); however, the LWPC effect was absent when four colors/words were used (Experiment 3). In Experiment 3, each word was paired with only two possible colors such that four separate word-color contingencies were present in each list, and high contingency responses could be predicted on the most frequent trial type within the mostly congruent and mostly incongruent lists. Logan et al. suggested that the manipulation exceeded capacity limitations; participants could not keep in mind the four word-color contingencies that existed within the list and so they abandoned the strategy. Such a finding is unanticipated by accounts that posit a list-wide strategy of filtering out words in the mostly incongruent list and fuller processing of (e.g., reading) words in the mostly congruent list. A word-filtering strategy should minimize interference in a mostly incongruent list even when a large number of color-word contingencies are present.

A number of recent studies have examined whether the LWPC effect reflects global, list-level modulation of word reading or the (possibly strategic) learning of contingencies or item-specific control. Bugg et al. (2008) determined whether a LWPC effect is observed when item-specific influences are controlled. They created two sets of items (words/colors). One set of items (e.g., GREEN and WHITE) established LWPC. For example, in the mostly incongruent list, these two items were presented 75% of the time in the incongruent color associated with the set. In the mostly congruent list, these two items were presented 75% of the time in the congruent color. Critically, a second set of items (e.g., RED and BLUE) was presented 50% of the time as congruent and 50% of the time as incongruent in *both* lists. Thus, these items were 100% identical and presented equally frequently in the mostly congruent and mostly incongruent lists. The key comparison for evaluating whether the LWPC effect reflected non-item-level processes was the magnitude of Stroop interference for the 50% congruent items in the mostly incongruent vs. mostly congruent list. Contrary to list-level control or strategic accounts, the LWPC effect was limited to the biased set of items (GREEN and WHITE) and was not obtained for the 50% congruent items that controlled for item-level influences.

Blais and Bunge (2010) used an almost identical design as Bugg et al. (2008) and replicated their primary result, again showing no evidence of list-level control. Moreover, Blais and Bunge had participants perform the Stroop task while in an fMRI scanner. They found that the anterior cingulate and dorsolateral prefrontal cortex, two regions previously implicated in top-down (e.g., list-wide) control (Botvinick et al., 2001), were selectively activated under conditions where item-specific control was presumed to operate (i.e., in contrasts involving the biased set of items). There were no differences in activation of these regions of interest in contrasts involving the 50% congruent items across the mostly congruent and mostly incongruent blocks. Like the findings of Bugg et al., these findings strongly challenged the view that list-level control is a mechanism underlying the LWPC effect.

Bugg and Chanani (2011) pursued the issue further by investigating whether the use of small stimulus sets precluded list-level control. When PC is defined by two-item sets, high contingency responses exist for congruent trials from the mostly congruent condition and incongruent trials from the mostly incongruent condition. Bugg and Chanani speculated that participants may not have engaged list-level control, a putatively more resource demanding process (cf. Braver et al., 2007), because they were capable of quickly and accurately performing the task using associative learning (prediction of high contingency responses) on the majority of trials within the mostly congruent and mostly incongruent lists. So they increased the number of items defining the PC lists, yet maintained the set size of the 50% congruent items at two. Using a picture-word Stroop task, birds, dogs, cats, and fish comprised the biased set and pigs and seals the 50% congruent set. Again, the key question centered on whether a LWPC effect would be obtained for the items in the 50% congruent set. In this study, unlike previous studies, that effect was in fact found, as was the LWPC effect for the biased set of items. Interestingly, the size of the LWPC effect (MC interference–MI interference) was larger for the biased set of items (62 ms), for which both item-specific and list-level mechanisms could be contributing, than the 50% congruent items (39 ms), for which only list-level control could be contributing. This suggests that the confound between ISPC and LWPC in most LWPC studies could inflate the size of the LWPC effect. The obtainment of an LWPC effect for 50% congruent items was also theoretically important in revealing conditions under which the LWPC effect reflects at least some contribution of list-level control and in developing a measure (i.e., the LWPC effect on 50% congruent trials) that permits researchers to selectively gage this control strategy.

A similar conclusion emerged from the work of Hutchison (2011). Like Bugg and Chanani (2011), Hutchison used items that were matched in congruency, however, the congruency was not 50%; instead he examined items that were 67% (mostly congruent) or 33% congruent (mostly incongruent) and which were embedded in mostly congruent or mostly incongruent lists. Additionally, for the mostly incongruent items, he varied whether or not items were associated with a single high contingency response. LWPC effects emerged when comparing interference across mostly congruent and mostly incongruent lists for each item type, but were strongest for mostly congruent items. This LWPC effect cannot be accounted for by item-specific influences and highlights the interaction of global control strategies with contingency learning processes. Collectively, the findings of Hutchison, and those of Bugg and Chanani, reinvigorated the list-level control account of LWPC effects and the idea that global strategies are sometimes used in resolving Stroop interference.

Following suit, Bugg et al. (2011b), sought converging evidence for the operation of list-level control using a slightly different method involving neutral trials (non-color words presented in different ink colors) that might be analogized to 50% congruent items in prior methods (Bugg et al., 2008; Blais and Bunge, 2010; Bugg and Chanani, 2011) in that they have no item-specific bias. Neutral trials are 100% neutral regardless of the overall bias of the list in which they reside. In their first experiment, neutral trials were embedded in mostly congruent, mostly incongruent, and mostly neutral lists, and six color-word stimuli were used so as to bias participants away from relying on associative/contingency learning (Bugg and Chanani). Mostly neutral lists were included to gain leverage on the question of what factors potentially trigger engagement of a list-level control strategy. Some models propose that the presence of a high degree of response conflict is a key determinant of top-down control processes used to minimize interference (e.g., Botvinick et al., 2001). It is possible, however, that list-level control is engaged whenever the irrelevant (word) dimension has little utility to responding, even when that dimension creates negligible response conflict (e.g., when most trials are neutral; cf. Melara and Algom, 2003). On this view, evidence for list-level control would be present in both the mostly incongruent and mostly neutral lists relative to the mostly congruent list. Here, such evidence would be a speeding of response times on neutral trials [in addition to reduced interference (i.e., incongruent-congruent RTs) in the same conditions]. Indeed, this is precisely what was found suggesting that a list-level strategy for attenuating interference such as word filtering was engaged in both the mostly incongruent and mostly neutral lists.

In a second experiment, Bugg et al. (2011b) used a third method for assessing the contributions of list-level control to the LWPC effect. Participants performed a Stroop task with an LWPC manipulation. However, they were also asked to perform a secondary prospective memory task during the Stroop task. Participants had to remember to press a response key (Stroop responses were vocal) whenever they encountered the word HORSE. In a control condition, participants pressed the response key whenever they encountered a particular pattern surrounding the Stroop stimulus. If participants implement a list-level word-filtering strategy in the mostly incongruent list, then performance on the secondary task should be impaired but only when the secondary task requires responding to a particular word and not when it requires responding to a particular pattern. As expected, less Stroop interference was observed in the mostly incongruent vs. mostly congruent list. This finding, however, did not adjudicate between item-specific and list-level processes because item-specific control, for example, could produce a similar pattern. Critically, the reduction in interference in the mostly incongruent list was accompanied by impairment in secondary task performance (relative to the mostly congruent list), and the impairment was specific to the word HORSE condition. The fact that the impairment was observed only for the word condition and not for the pattern condition was important in ruling out accounts of the impairment based on the difficulty of the ongoing Stroop task, which some might argue is higher when most trials are incongruent. These results further support the role of a list-level control strategy that modulates word reading even prior to stimulus onset; it is unclear how an item-specific mechanism that acts post-stimulus onset would account for the pattern of findings on neutral trials across the two experiments (Bugg et al., 2011b).

### Context-specific proportion congruent manipulations

A different approach to evaluating the item-specific control and contingency learning accounts stems from a third category of PC manipulations, termed CSPC manipulations. Here, PC is varied between different contexts in which the same items are presented. If features of an item can rapidly trigger attentional filters tailored to processing of particular items, then environmental cues that are associated with particular items, such as the location context in which an item appears, may also act as stimuli for triggering rapid-online control over attentional filtering. In a seminal study, Crump et al. (2006) used a prime-probe version of Stroop. A word (prime) was presented at fixation followed by a congruent or incongruent color patch (probe) that appeared randomly above or below fixation. The location of the color patch defined the context for the PC manipulation. For example, probes appearing above fixation were mostly congruent (75%) and probes appearing below fixation were mostly incongruent (75%). As with ISPC procedures, LWPC was 50/50 congruent and incongruent. Here again, Stroop effects were larger for probes appearing in the mostly congruent than mostly incongruent locations. Such CSPC effects have also been observed in a more traditional Stroop paradigm using font, rather than location, as the contextual cue (Bugg et al., 2008). Importantly, in these designs, all word-color pairs were presented with equal frequency and rule out accounts based on stimulus-response learning (e.g., associative/contingency). However, even in these designs an event-frequency learning process sensitive to unique word-color-location (or word-color-font) compounds could account for the observed CSPC effects.

Crump and Milliken (2009) addressed the event-frequency confound by manipulating PC both at the context and item-level. Two item-types were defined: context and transfer items. Context items carried the PC manipulation and were necessarily frequency biased. For example, red and green Stroop items were 100% congruent when they appeared above fixation and 100% incongruent when they appeared below fixation. Transfer items were not frequency biased. For example, yellow and blue items were 50% congruent and 50% incongruent in both locations. Both context and transfer items were mixed together and presented randomly in both locations within each block of trials. The question of interest was whether the attentional filter applied to the context items in their respective locations would generalize to the frequency-unbiased transfer items appearing in those locations. Indeed, CSPC effects were observed for the transfer items, with larger Stroop effects for transfer items when they appeared in the mostly congruent vs. mostly incongruent contexts. This transfer effect provides a clear example of rapid, online, context-triggered control adjustments.

## Parallel Developments in the Flanker Literature

The issues raised by PC research in the Stroop literature apply across selective attention tasks. A common feature of attention tasks is that they present participants with information selection problems. The Stroop task measures ability to select word from color information. The flanker task measures spatial attention ability to select central and ignore distracting peripheral information (Eriksen and Eriksen, 1974). Typically participants identify a target (e.g., a T or L) that is flanked by compatible (T T T) or incompatible (LTL) distractors. Responses are faster for compatible than incompatible trials indicating a failure of distractor suppression. As in the Stroop task, modulations to the size of the flanker effect measure processes that adjust attention filters (albeit spatial filters) to enhance or suppress processing of peripheral information. Thus, the flanker paradigm offers an opportunity to investigate how various forms of control coordinate spatial attention.

List-wide proportion congruent, ISPC-like and CSPC manipulations similar to those applied in Stroop have been shown to control the size of the flanker effect (e.g., Miller, 1987; Cohen et al., 1999; Corballis and Gratton, 2003; Lehle and Hübner, 2008; Wendt et al., 2008). As is the case for the LWPC manipulation in Stroop, the same manipulation in flanker tasks produces a similar pattern with larger compatibility effects for mostly compatible than mostly incompatible lists (e.g., Gratton et al., 1992; Lehle and Hübner, 2008, Experiment 2 Training Block performance; Taylor, 1977, Experiment 2; Wendt and Luna-Rodriguez, 2009). Unlike in Stroop, however, researchers have yet to examine whether a list-level strategy makes any contribution independent of item-specific influences (e.g., item-specific control or contingency learning). One possible reason this line of investigation has not been pursued is because ISPC designs and effects have not received much focus in flanker tasks and there has not been an empirical challenge to list-level explanations. This is a ripe area for future investigation.

In the realm of CSPC manipulations, by contrast, much work has been done exploring the flanker task. For example, Corballis and Gratton (2003) presented flanker items in different location contexts correlated with different levels of PC. Larger compatibility effects were observed for mostly congruent than mostly incongruent contexts. The locations were to the left and right of fixation because they were interested in determining whether cognitive control processes could become lateralized across hemispheres. Their hemispheric control hypothesis assumes that each hemisphere is capable of representing distinct attentional sets for controlling information specific to the processing demands of information presented to each hemisphere. CSPC effects in flanker tasks have been shown for up to four unique locations (Wendt et al., 2008), indicating a rapid and flexible engagement of attentional settings depending upon the location in which an item appears.

An important question that has been addressed in CSPC studies concerns the types of contextual cues that are effective in producing context-triggered control adjustments. In the initial study of Crump et al. (2006), a striking asymmetry was observed such that location but not shape-based contextual cues (i.e., whether the color patch probe was a square or circle) produced CSPC effects (for a similar pattern, see Crump et al., 2008). The fact that location-based cues may be processed automatically (Logan, 1998) offers one explanation for the asymmetry. Context-triggered control adjustments are presumed to occur very rapidly post-stimulus onset, and subtle differences in the speed with which the context is identified (i.e., location is faster than shape) could drive which cues are useful signals of PC. Crump et al. (2008) tested another explanation, the relevance hypothesis, which proposes that a particular contextual cue will be effective to the extent that is relevant to the current task, and thus attended (Nissen and Bullemer, 1987). Crump et al. speculated that location-based information, although nominally as irrelevant as shape in their prime-probe Stroop paradigm, might generally receive greater attention due to the importance of orienting to location in order to identify other stimulus attributes (e.g., name color). To test their hypothesis, shape was made relevant by asking participants to count the number of probes that were squares (or circles) while performing the Stroop task. Initial support for the relevance hypothesis was obtained. The shape-based cue, which was previously ineffective in triggering context-specific adjustments in control, produced a CSPC effect when attention was directed to the shape dimension.

Lehle and Hübner (2008) examined whether another identity-based cue, color, would produce a CSPC effect. They used a flanker task that included numerals as stimuli, and participants judged whether the central target was odd or even. Stimuli presented in one color (e.g., green) were 80% congruent while stimuli presented in the second color were 20% congruent. A 50 ms compatibility effect was obtained but the magnitude of the compatibility effect did not vary as a function of whether the stimuli appeared in the mostly congruent or mostly incongruent color. The results of their first experiment, thus, supported those of Crump et al. (2006, 2008), Experiment 1b) in showing an absence of an identity-based CSPC effect. In a second experiment, however, Lehle and Hübner obtained the effect. The primary change was that participants initially completed a set of training blocks wherein they experienced a fixed association between stimulus color and PC. The goal was for participants to learn the association between green and 80% congruent, for example, and red and 20% congruent prior to performing the CSPC task, where green and red stimuli were randomly intermixed. The size of the compatibility effect was similar to Experiment 1 (54 ms); however, the effect was modulated by CSPC with a smaller effect observed for stimuli presented in the mostly incongruent color.

Color-based CSPC effects in the flanker task could depend on the existence of pre-learned associations between color and PC; however, the findings of Vietze and Wendt (2009) challenge this view. A letter-based version of the flanker task (e.g., SSHSS) in which stimuli were presented in yellow or green was used. One color was associated with a high (or low, in separate blocks) level of PC and the other was 50% congruent. Interference was reduced for the stimuli associated with a low as compared to high PC. This suggests that a color-based CSPC effect can be obtained without any prior training on the associations between color and PC. An alternative explanation is that context-level and list-level control were both at play. When only one color within a block is biased (high or low PC level) and the other is 50% congruent, the overall list has a slight bias (e.g., 64% congruent or incongruent), unlike the lists in typical CSPC paradigms (e.g., Crump et al., 2006, 2008; Lehle and Hübner, 2008; Crump and Milliken, 2009). As such, it is possible that part of the reduction in interference that was observed for mostly incongruent colors involves use of advance information (to alter attention) regarding the likelihood of interference within the list. Of course, if list-level control were making a robust contribution, one would have expected a reduction in interference for the 50% congruent color when paired with a mostly incongruent color as well (cf. Bugg and Chanani, 2011), and that was not found.

Finally, researchers have also examined whether temporal information can serve as a contextual cue for cognitive control adjustments. At least one study has found that temporal information, such as the duration of a fore period (200 vs. 1200 ms) preceding stimulus onset, is an effective cue for carrying the CSPC manipulation (Wendt and Kiesel, 2011).

As in the Stroop task, researchers using priming procedures similar to the flanker task have attempted to rule out explanations of the CSPC effect pertaining to stimulus-frequency. Heinemann et al. (2009, Experiment 1) employed a similar procedure as Crump and Milliken (2009), examining whether context-specific control adjustments would be observed for a set of frequency-unbiased items (presented equally often in both contexts). They used a prime-target paradigm in which the “flanker” preceded the target, appearing in the same location as the target, rather than flanking it. Judgments of whether the target was smaller or larger than five were made on compatible (e.g., prime = 7 and target = 6) and incompatible (e.g., prime = 7 and target = 4) trials in the presence of a colored rectangle. The color of the rectangle was associated with the PC level of the accompanying stimulus. Contrary to a frequency-based account, the compatibility effect was smaller for frequency-unbiased stimuli that were accompanied by the mostly incongruent color than the mostly congruent color.

Heinemann et al. (2009) examined the role of conscious awareness in the obtainment of the CSPC effect by weakly (Experiment 1) vs. strongly (Experiment 2) masking the prime. The context manipulation did not produce differential compatibility effects when the prime was strongly masked. The authors concluded that conscious access to the incompatible prime stimuli is necessary for context-triggered adjustments in cognitive control, possibly because access allows participants to determine the prime’s (distractor’s) utility to processing the target information, which allows modulation of attention to the prime on subsequent trials. Note that at first blush this may seem discrepant with findings that show participants do not have conscious access to the PC manipulation (i.e., cannot report the approximate proportion congruence for each context, Crump et al., 2006). However, a subtle but important difference is that even when primes are only barely visible, this information may be sufficient for participants to develop an implicit sense of PC even if they cannot consciously report the identity of the prime.

Most recently, in a fMRI study, King et al. (2012) examined the neurophysiological underpinnings of CSPC effects in a variant of flanker that used face-stimuli as targets and distractors, and location as the cue to signal PC. Their task used unique faces on every trial and thus ruled out S-R learning as an explanation for their observed CSPC effects. Context-specific modulation of flanker interference was tied to activity in the medial superior parietal lobule that displayed functional coupling with visual regions processing the flanker stimuli. They also showed that CSPC effects depended on context repetitions across trials, and suggested that context cues may not trigger online retrieval of attention settings, but instead may instantiate or prime attentional sets that apply forward when the context repeats.

## Parallel Developments in the Task-Switching Literature

Task-switching costs – the finding that performance costs ensue when switching rather than repeating a task– are influenced by a range of processes from higher-level preparatory and strategic processes to lower-level cue-encoding and priming processes. Task-switching costs are influenced by list-wide proportion manipulations. Note, however, that these manipulations do not center on PC but instead on proportion repeat. Task-switch costs are larger for high proportion task-repeat than for low proportion task-repeat blocks of trials (Dreisbach et al., 2002; Dreisbach and Haider, 2006; Schneider and Logan, 2006). Task-switching costs are also influenced by item-specific proportion repeat manipulations (Leboe et al., 2008). Task-switch costs are larger for items associated with a high than low proportion of repeats. Task-switching costs can also be influenced by contextual cues that are predictive of particular tasks (Mayr and Bryck, 2005, 2007; Rubin and Koch, 2006). For example, task-switching costs are reduced when tasks appear in predictive contexts (such as location) rather than in unpredictive contexts. These findings are very much in line with the idea that stimulus information can retrieve attentional control settings and apply them to adjust online performance. In this case task-sets, rather than spatial attention or word-filtering settings, are retrieved by contextual cues.

Somewhat more abstractly, task-switching costs are also influenced by context-specific proportion repeat manipulations (Leboe et al., 2008; Crump and Logan, 2010). Task-switch costs are larger in the context associated with a high than low proportion of task-repeats. In these cases, the contextual cue was not associated with a particular task, but instead associated with likelihood of switching a task. One interpretation of this latter context effect is that contextual cues can retrieve a signal that controls whether or not a recently used task-set is retrieved and applied to current performance.

## Control Processes and Representations Involved in LWPC, ISPC, and CSPC Effects

To take stock, PC effects can take several forms (list-wide, item-, and context-specific), they are highly robust, and they have been replicated both within and across paradigm boundaries in attention. For these reasons, we view PC manipulations as a useful tool to better understand cognitive control processes in general. Earlier, we forwarded the attention and action theory (Norman and Shallice, 1986) as a tool to better classify levels of cognitive control. We distinguished between proximal and distal forms of control and endogenous and exogenous forms of control. In this section we describe how different PC effects provide insight into these forms of control.

### Supervisory control

Supervisory control refers collectively to those processes engaged in strategic, endogenous, anticipatory, preparatory, proactive, executive, or voluntary control. Supervisory control reflects operations of the elusive homunculus, where the intentions, plans, and strategies voluntarily adopted by a performer direct, guide, and coordinate how attention selects information in the environment. Supervisory processes comprehend task-instructions and set over-arching goals for task performance like speed-accuracy tradeoffs, attention to task-relevant information, and application of task-specific rules. Supervisory control monitors ongoing performance and makes adjustments to the activation of action plans when performance runs amok. Supervisory control is endogenous and distally acts on the proximal control units that direct attention and action. Similarly, in task-specific models of attentional selection, like Stroop (Cohen et al., 1990, and in domain-general models of attention (Bundesen, 1990; Logan, 2002), the setting of weights that filter perceptual information is assumed to be under supervisory control. The setting of weights is an endogenous act of control, and the weights themselves refer to the proximal mechanisms that filter information.

Supervisory processes are often invoked to explain LWPC effects (e.g., Lowe and Mitterer, 1982). If participants become aware of the manipulation, then they have every opportunity to anticipate and prepare for upcoming congruent or incongruent trials. In the context of Stroop, this would imply that participants are capable of voluntarily adjusting the extent to which they suppress word information (Cheesman and Merikle, 1986; Balota and Faust, 2001). This is plausible, as Raz et al. (2006) demonstrated that highly suggestible subjects show reduced Stroop interference when they are told to imagine words as not word-like. Strategic control also requires effort to maintain attentional focus over the course of the task, and it is well known that PC effects vary with working memory capacity, presumably reflecting the fact that people low in working memory capacity fail to consistently maintain the attention settings required for their adopted strategy (Kane and Engle, 2003; but see Hutchison, 2011, for evidence of larger LWPC effects for individuals with low working memory capacity).

Strategic control accounts assume awareness of the PC manipulation, however participants may show list-wide effects even when unaware of the manipulation. Blais et al. (2012) found that shifts in cognitive control across mostly congruent and mostly incongruent lists largely reflected implicit knowledge of PC. Participants who were more aware of the LWPC manipulation were not more likely to show a significantly larger LWPC effect, as might be expected if participants were using a voluntary strategy based on awareness of PC. An alternative idea is that of Melara and Algom (2003) who refer to attention being differentially drawn to the irrelevant dimension in mostly congruent vs. mostly incongruent conditions depending on the irrelevant dimension’s utility to responding. Note that no reference to voluntary filtering of the irrelevant dimension is made per this very viable account. Instead, the idea is that this biasing of attention occurs relatively automatically when correlations are present between the relevant and irrelevant dimension, as is the case when PC is manipulated [cf. Kinoshita et al.’s 2011, explanation of the effects of list-wide congruency proportion on priming (with visible primes) that refers to *implicit* tracking of the prime’s utility in predicting the response].

Item-specific proportion congruent and CSPC manipulations typically do not invoke voluntary control, whereby participants become aware of the associations between particular items or contexts and their likelihood of congruency, and then use this knowledge to prepare for upcoming trials. Most ISPC designs do not probe awareness of the ISPC manipulation so it is not clear whether participants have explicit knowledge of the manipulation. In an ISPC-like paradigm, Schmidt et al., (2007 found that evidence of the learning of four non-color-word contingencies was present even in participants who were explicitly unaware of the contingencies. Given that ISPC designs have many different items (usually between 4 and 8), it seems unlikely that participants would become aware of all of the item-specific associations.

It is perhaps easier to imagine that voluntary control could account for CSPC effects (cf. Heinemann et al., 2009). Many CSPC designs employ two contexts: high and low PC. It is possible that participants become aware of the CSPC manipulation and simultaneously prepare two attentional sets, one for each context. On this view, CSPC effects would reflect rapid voluntary switching of attentional set in response to contextual information. However, participants are unable to explicitly report the proportions of congruent and incongruent items in the high and low PC contexts (Crump et al., 2006). In the same set of studies, CSPC effects were observed for location cues, but not for shape cues. Following up, Crump et al. (2008) attempted to make shape cues effective by informing participants about the CSPC manipulation. Participants signed a consent form indicating they were aware of which shapes signaled high and low PC, however CSPC effects were not observed. Interestingly, at the end of the experiment participants were again probed for their knowledge of the manipulation, and at this time they failed to report the correct proportions. This leaves open the possibility that the awareness manipulation was not strong enough, however it also underscores that participants become absorbed with the task and lose knowledge of the CSPC manipulation by the end of the task.

### Stimulus-driven control

Stimulus-driven control refers to exogenous cuing or triggering of proximal representations coordinating attention and action. Stimulus-driven control covers, but is not limited to, classic automatic influences that occur in a rapid, non-voluntary, and stereotyped or inflexible manner. Well-known examples in attention include interference from distracting word or spatial information in Stroop or Flanker, attention capture by salient perceptual information (Theeuwes, 1991, 1992), or peripheral visual cuing effects (Posner and Cohen, 1984). Stimulus-driven control also guides action in the context implicit sequence learning tasks (Nissen and Bullemer, 1987), and broadly covers classical conditioning phenomena in human and animal learning (Pavlov, 1927; Rescorla and Wagner, 1972). In these examples stimuli are assumed to capture attentional resources or retrieve learned responses.

Stimulus-driven control processes are invoked to explain ISPC and CSPC effects. Here features of the item or the item’s context act as environmental cues. Cues trigger associated representations controlling attention and action. In the Norman and Shallice (1986) theory stimuli retrieve action schemas. In the PC literature stimuli are assumed to retrieve associated responses or attentional settings (Jacoby et al., 2003). As we have discussed, a major debate in the PC literature aims to adjudicate between contingency learning accounts that invoke stimulus-response associations, and stimulus-driven attentional control accounts that invoke stimulus-attention associations in accounting for ISPC and CSPC effects. These representational issues are summarized in the next section. Aside from this debate, there is broad consensus that stimulus-driven processes play an important role in PC effects, and in guiding attention and action in general.

### Low and high levels of proximal control

Proximal control refers to those representations that are exogenously or endogenously brought to bear in the direct control of attention and action. The stimulus-response association is perhaps the most classic example of a representation controlling action. There is broad consensus that stimulus-response associations are a fundamental building block of performance across attention and action, and there is wide recognition that these representations mediate some, but not all PC effects.

One of the major new insights into proximal control provided by the PC literature could be termed the *stimulus-attention association*. Conventionally, stimuli are assumed to be directly associated with responses, whereas attentional settings are assumed to be controlled by supervisory processes, and not associated with or triggered by environmental cues. The stimulus-attention association allows for the possibility that environmental cues can be associated with and trigger the application of attentional filters that, like responses, have been paired together during a learning experience. This kind of representation has been invoked to explain ISPC and CSPC effects, whereby the features of the item or contextual features rapidly trigger associated attentional filters that modulate the size of congruency effects.

Stimulus-response and stimulus-attention associations point to different levels of proximal control. Stimulus-response associations can be considered as low-level proximal control. Low-level refers to the notion that stimulus-response associations are well-learned, inflexible or stereotyped responses. Once triggered they proceed with minimal internal ability to make adjustments to action. The possibility of stimulus-attention representations suggests a more flexible, higher level of proximal control. Whereas stimulus-response associations can be highly stereotyped with stimuli retrieving only those specific actions paired in the past, stimulus-attention associations can allow for flexible control by triggering generalizable attentional filters that allow selection processes to transfer across items.

The PC literature has developed precise methods to identify effects that show evidence for stimulus-attention representations, but it is not alone in providing supporting evidence. Contextual cues can direct attention toward target location in visual search (Chun and Jiang, 1998). Long-term negative priming (DeSchepper and Treisman, 1996; Grison et al., 2005), long-term inhibition of return effects (Tipper et al., 2003), long-term aftereffects in the stop-signal task (Verbruggen and Logan, 2008), and long-term priming of pop-out effects in attention capture (Thomson and Milliken, 2012a,b) show that stimuli can retrieve attentional filters applied to them from the recent and distant past. Last, prior experience with viewing natural scenes controls eye-movements and sampling of information from familiar images (Ryan et al., 2007). All of these examples demonstrate stimulus-driven control over a variety of attention processes and further support a distinction between low and high levels of proximal control.

The nature of stimulus-attention representations are currently not well understood. Crump and Milliken (2009) forwarded an episodic account whereby memory encodes the stimulus, response, contextual features, and attentional procedures or filters employed during performance. In this way, contextual information in the task environment can cue retrieval of attentional settings used in the past and apply them to control online processing in the present. This account acknowledges stimulus-driven control as a process that guides selective attention. As well, the account assumes enriched memory representations that not only code stimulus-response information, but also code the history of attentional operations that have been applied during performance. In this way, the account is similar to the event-files account Spape and Hommel (2008) forwarded to explain the selectivity of sequential modulations of the auditory Stroop effect. The modulations were limited to sequences in which the to-be-ignored word (“high” or “low”) was spoken by the same voice on trial *n* − 1 and trial *n*. Speaker voice was apparently a (contextual) feature to which the attentional control operations associated with trial *n* − 1 were bound (along with actions, etc.). Only when the same voice was spoken on trial *n* were the attentional operations reactivated, leading to a performance benefit.

A critical as yet untested assumption of these accounts is that the attentional filters triggered by stimuli are themselves bound together in records of prior experience (e.g., in an episode or event file) that code stimulus, response, and attentional filtering information. With respect to the attention and action theory, this assumption is akin to saying that the contention scheduling system codes more than stimulus-response units, it also codes stimulus-attention units or perhaps stimulus-attention-response units. An alternative possibility is that attention filters are not bound in a long or short-term episodic record, but that they trigger application of attentional sets maintained outside of contention scheduling. Part of the distinction rests on whether the stimulus-triggered adjustment occurs during retrieval of bound attentional filters that are integrated into online attentional sets, or whether multiple attentional sets are maintained online and stimuli bias application of existing attentional sets. This distinction is made apparent in differing computational accounts of PC effects that are described in the next section.

## Computational Accounts

List-wide and item-level PC effects have been discussed in more formal computational models. The aim of these models fits well with the aims of abandoning the conventional controlled vs. automatic dichotomy, and more precisely defining the processes underlying PC effects and cognitive control in general. Perhaps the most well known of extant computational models of PC effects is the conflict-monitoring account (Botvinick et al., 2001). According to this account, the anterior cingulate acts as a conflict-monitor, a cumulative recorder of conflict on all preceding trials. When conflict is frequent, as in a mostly incongruent list, the anterior cingulate signals regions such as dorsolateral prefrontal cortex to increase its top-down influence on performance. The notion of a conflict-monitoring system also challenges the controlled vs. automatic dichotomy as the monitoring system enacts control on an automatic basis driven by conflict signals. The control is endogenous in the sense that its origin stems from the processing of conflict signals, and these signals feed global, top-down adjustments buffering against conflict in the immediate future. Botvinick et al. characterize this global influence as heightened processing of the relevant dimension (see also Egner and Hirsch, 2005), though accounts positing heightened top-down filtering of the word dimension are equally viable (Lindsay and Jacoby, 1994). Conflict-based adjustments are partly distal in the sense that the monitoring system acts on the attention processes filtering the relevant or irrelevant dimension, and they are partly proximal in the sense that there is a kind of closed processing loop between conflict detection and subsequent adjustment.

The conflict-monitoring account successfully anticipates the LWPC effect, including the finding of this effect for congruency-matched items (e.g., 50%, Bugg and Chanani, 2011; 67 or 33%, Hutchison, 2011) and neutral trials (Bugg et al., 2011b). In addition to explaining the LWPC effect via a global heightening of top-down control in mostly incongruent lists, the conflict-monitoring account might also explain the effect via a reactive heightening of control, otherwise known as conflict-adaptation. LWPC manipulations necessarily bias the number of trials that are preceded by an incongruent trial. For example, in mostly incongruent blocks, most trials are preceded by an incongruent trial. This leaves open the possibility that smaller congruency effects in mostly incongruent vs. mostly congruent blocks are driven by conflict-driven adjustments on a local, trial-to-trial basis. Such an explanation may be less likely to account for LWPC effects on congruency-matched items or neutral items, however, when such items do not share overlapping features (relevant or irrelevant) with the items that establish the bias of the list (i.e., those items that are presented as incongruent on 75% of trials in a mostly incongruent list). Conflict-adaptation effects are more fickle under such conditions (e.g., Mayr et al., 2003; Nieuwenhuis et al., 2006).

The conflict-monitoring account has difficulty accounting for ISPC and CSPC effects. Most ISPC and CSPC designs balance congruent and incongruent items at the list-wide level. In other words, conflict occurs on 50% of trials within a list, and the degree of conflict present in a given list is equivalent for all items. The finding of differential interference effects for different items or contexts is therefore difficult to reconcile with a model that posits a global level of top-down control across the list. In addition, in the case of ISPC and CSPC manipulations, the number of trials that follow incongruent items is also balanced such that it is unlikely that sequential effects such as conflict-adaptation can account for the corresponding effects. As a case in point, Crump et al. (2006) reported a sequential analysis in their CSPC design. They found significant sequential effects – the Stroop effect was smaller for trials following incongruent than congruent trials – however, these did not interact with the CSPC effect (see also Vietze and Wendt, 2009, for a similar pattern in their study on color-based CSPC effects in the flanker task).

Several researchers have recently forwarded computational models that include item-specific mechanisms in an attempt to provide alternatives to the conflict-monitoring model. For instance, Blais et al. (2007) proposed an item-specific conflict-monitoring account of ISPC effects. The model assumes that conflict signals are item-specific rather than general. The model also assumes a role for online maintenance of multiple, item-specific attentional sets, and thus does not assume that online-adjustments are driven by memory retrieval processes. When conflict is frequently experienced for a given item (e.g., GREEN is shown frequently in white), control adjustments are made only to the relevant pathway for the specific item generating the conflict (e.g., attention to the color white is boosted for the word GREEN). Mostly incongruent items frequently cause conflict, which in turn triggers conflict-induced adjustments for those items, leading to smaller interference effects relative to mostly congruent items. Recent findings showing that item-specific control, when dissociated from contingency learning mechanisms, has a selective or more pronounced influence on incongruent trials (Bugg et al., 2011a; Bugg and Hutchison, 2012) lend support to the idea that conflict plays a role in triggering item-specific adjustments. Interestingly, this pattern is not consistently observed in CSPC paradigms that index context-specific control (e.g., Crump et al., 2006), which raises the question of whether context-specific control adjustments are triggered by the occurrence of conflict. It is possible that access to the contextual cues, perhaps especially in the case of location, occurs sufficiently rapidly such that control adjustments are triggered prior to the detection of conflict. This remains to be explored in future modeling efforts.

Another open question is how the item-specific conflict-monitoring model accounts for transfer in ISPC and possibly CSPC paradigms. If the model boosts control only for the specific item (word-color compound) producing conflict, then transfer would seem unlikely given that transfer trials typically include an old word paired with a new color (or picture) or an old color (or picture) paired with a new word (e.g., Crump and Milliken, 2009; Bugg et al., 2011a; Bugg and Hutchison, 2012).

An appeal of the model of Blais et al. (2007) is that it successfully models not only ISPC but also LWPC effects. However, with regard to the latter, it is important to note that the LWPC effects that were modeled were confounded with ISPC effects. Thus, it is uncertain whether the item-specific conflict-monitoring model can account for more “pure” indicators of list-level control such as LWPC effects for congruency-matched items (Bugg and Chanani, 2011; Hutchison, 2011). It seems rather unlikely, given that the congruency-matched items are identical in the mostly congruent and mostly incongruent lists (Bugg and Chanani; Hutchison). Because conflict is identical for these items across lists, similar levels of interference should be observed according to the model. Similarly, it is unlikely that conflict-adaptation effects resulting from item-specific conflict can account for the LWPC effect observed for congruency-matched items. In the study of Bugg and Chanani, congruency-matched items shared no features (relevant or irrelevant) with the items that established the bias of the list. According to the item-specific conflict-monitoring model, such adjustments would influence performance on the subsequent trial only when the word repeats (Blais et al., 2007), and the word never repeated when transitioning from a high conflict (biased) to a 50% congruent item (see also Hutchison, 2011). By contrast, another model that includes an item-specific component, here one that reflects a conflict modulated Hebbian learning rule, accommodates both item-specific and item-non-specific adaptation (e.g., sequential effects on non-repeating items; Verguts and Notebaert, 2008). As such, it accounts for the ISPC effect. Additionally, it might accommodate the LWPC effect for congruency-matched and neutral items if the sequential effects on the non-repeating trials in the paradigms that have revealed these LWPC effects are sufficiently robust. The model does, however, require an additional assumption to account for such effects, namely that there is a little bit of carry-over of the top-down control settings from trial *n* − 1 to trial *n*.

## Open Issues for Future Research

The already rich PC literature might be expanded in several theoretically important ways that would enhance our understanding of the many faces of cognitive control. For instance, PC designs involve learning about stimulus-response and stimulus-attention associations. It remains unclear whether these associations are formed by the same learning processes, and are established at the same rate. There is some evidence to suggest they may not be. For example, Jacoby et al. (2003) found that the contingency-confounded ISPC effect was present within just 16 trials. By contrast, Crump et al. (2006) and Bugg et al. (2008) found that their CSPC effects, believed to reflect stimulus-attention associations, developed more slowly, and in some cases these effects interacted with block (i.e., were not observed in initial blocks but grew stronger with time).

Second, PC effects have been studied in single session designs where the learning occurs inside the experimental session. It remains unclear whether these learning experiences establish long-term associations that would continue to influence performance a day or a month in the future, similar to the types of effects that have been observed in other attention paradigms (e.g., see DeSchepper and Treisman, 1996, for evidence of negative priming at a 1 month interval). Similarly, stimulus-attention associations may be useful for particular items and contexts in specific situations, and new stimulus-attention associations may be required when task demands change at the item or context level. The time-course with which old stimulus-attention associations interfere with learning of new stimulus-attention associations remains an open question for future research.

A third open issue concerns the experience of conflict and the formation of associations. There is some evidence that learning about PC not only depends on item-frequency, but also depends on experiencing conflict during the learning experience. For example, Crump et al. (2008) showed a location-based CSPC effect in a Stroop color-naming task with word primes and color patch probes, but CSPC effects were not observed when the task was reversed (word naming with color patch primes and word probes). The word-naming version of the Stroop task reduces the experience of conflict, and suggests that conflict may play an important role in the learning of stimulus-attention associations.

Broadly speaking, we have endorsed the view that ISPC and CSPC effects reflect stimulus-driven control whereby item or context-level cues trigger attentional adjustments. This shows that stimulus-attention associations can be tailored for specific items, and for classes of items that appear in similar contexts. The principles guiding reliance on item- or context-specific stimulus-attention associations remain unclear, however. For example, as noted above, it is not certain whether item and context-level control adjustments are both conflict triggered, nor is it clear when item-level associations would dominate over context associations or vice versa. Bugg et al. 2008) found that when words (an item-level signal) and font (a contextual signal) were correlated with PC, the PC effect was no larger than when words independently signaled PC, which might be interpreted as preliminary support for the dominance of item-specific signals of control.

Another issue is that PC designs, especially those examining manipulations other than LWPC, have largely been carried out in behavioral paradigms (but see Blais and Bunge, 2010; King et al., 2012). The neural substrates coding stimulus-attention associations remain unspecified. Imaging studies may be particularly advantageous for examining whether control is indeed anticipatory/preparatory in the context of LWPC manipulations vs. stimulus-driven in the context of ISPC and CSPC manipulations. Methodologies are available that permit the examination of sustained activation patterns, which one would expect to accompany list-level control, and transient activation patterns, which should characterize the item- and context-level adjustments. Moreover, it would be advantageous to examine the time-course of LWPC, ISPC, and CSPC effects. Cleary, the latter two reflect very rapid control adjustments, but it is not certain whether the time courses differ for ISPC and CSPC effects, or whether the trigger for such adjustments is a perceptual feature (e.g., shape differences such as font, Bugg et al., 2008) as opposed to the conflict associated with processing of the irrelevant dimension. These questions could be addressed using event-related potentials.

Finally, there is much room for application of the range of PC manipulations to other tasks in which it would be theoretically advantageous to isolate voluntary control from stimulus-driven mechanisms, and vice versa. There is evidence of LWPC effects in the Simon task (e.g., Hommel, 1994; Toth et al., 1995), which shares some features with the Stroop and flanker tasks to which we devoted much attention. Determining whether PC manipulations are useful for examining cognitive control in tasks that are quite different from Stroop and flanker (e.g., task-switching; Go No-Go) is a necessary next step in evaluating whether the levels of control concepts presented herein might have broader appeal.

### Final thoughts

People learn to optimize their performance in a complex and unruly world. It is increasingly clear that multiple levels of control guide performance during and after learning. We have distinguished broadly between endogenous vs. exogenous and proximal vs. distal aspects of control. Endogenous control highlights opportunity for volition to guide performance, and exogenous control highlights opportunity to offload control to the environment. Both endogenous and exogenous control act distally on the many kinds of proximal control representations that allow attention to filter relevant from irrelevant information and the motor system to guide action. The relationships between levels of control and the extent to which different control processes contribute to performance are not well understood.

On the one hand, investigating multiple levels of control calls for researchers to study each level independently. This involves a terminology of control that permits fine distinctions between levels and experimental rigor to create process pure measurements of each process. The PC literature is an illustration of the difficulty in achieving this rigor. Nevertheless, such an approach could potentially answer quantitative questions about control-based adjustments. For example, do voluntary, conflict-based, or stimulus-driven forms of adjusting attention all have the same power to change attentional settings? The approach might also encourage researchers to experimentally disentangle contributions of stimulus-response, stimulus-attention, conflict-based, and voluntary processes potentially mediating effects of interest.

On the other hand, it would be unfortunate if by dividing-and-conquering the territory of cognitive control it would be left without some theme of unification. Investigating multiple levels of control also calls for researchers to clarify relationships between levels. Do different forms of control act on the same proximal mechanisms of control? Do these forms of control operate exclusively from each other or are they interrelated over the course of learning? For example, are stimulus-attention associations formed strictly by incidental or implicit learning processes, or do they reflect a learning process that associates voluntary control of attention together with particular cues eventually mediating stimulus-driven control? We encourage both approaches, investigating the nuances of different forms of control and how they act in concert as paths toward understanding the manifold ways that cognitive control coordinates performance.

## Conflict of Interest Statement

The authors declare that the research was conducted in the absence of any commercial or financial relationships that could be construed as a potential conflict of interest.
